# Differential Effects of Normoxic versus Hypoxic Derived Breast Cancer Paracrine Factors on Brain Endothelial Cells

**DOI:** 10.3390/biology10121238

**Published:** 2021-11-27

**Authors:** Mariam Rado, Brian Flepisi, David Fisher

**Affiliations:** 1 Medical Bioscience Department, Faculty of Natural Sciences, University of the Western Cape, Robert Sobukwe Road, Bellville 7535, South Africa; 3580480@myuwc.ac.za; 2 Department of Pharmacology, Faculty of Health Sciences, University of Pretoria, 9 Bophelo Road, Pretoria 0002, South Africa; brian.flepisi@up.ac.za

**Keywords:** breast cancer, cancer secretome, brain endothelial cells, mitochondrial activity, blood-brain barrier

## Abstract

**Simple Summary:**

The potential of breast cancer to spread to the brain increases the clinical complications of the disease; breast cancer is considered to have the second-highest capacity to spread to the brain after lung cancer. The brain is protected by highly specialized endothelial cells, forming a barrier against the entry of circulating molecules and cells. The ability of breast cancer cells to penetrate the protective endothelial barrier is still not completely understood. Here, we aimed to investigate the effect of breast cancer cells on the brain’s endothelial cells. We showed that breast cancer cells induce changes in endothelial cells by releasing factors that target the mitochondria, affecting the endothelial cells and their attachment to each other and, therefore, their function as a protective barrier of the brain. Understanding the mechanism that breast cancer cells utilize to affect endothelial cells under normoxic and hypoxic conditions contributes to the development of treatments to prevent the metastasis of cancer cells to the brain.

**Abstract:**

**Background**: The blood-brain barrier (BBB) is a central nervous system protective barrier formed primarily of endothelial cells that regulate the entry of substances and cells from entering the brain. However, the BBB integrity is disrupted in disease, including cancer, allowing toxic substances, molecules, and circulating cells to enter the brain. This study aimed to determine the mitochondrial changes in brain endothelial cells co-cultured with cancer cells. **Method**: Brain endothelial cells (bEnd.3) were co-cultivated with various concentrations of breast cancer (MCF7) conditioned media (CM) generated under normoxic (21% O_2_) and hypoxic conditions (5% O_2_). The mitochondrial activities (including; dehydrogenases activity, mitochondrial membrane potential (ΔΨm), and ATP generation) were measured using Polarstar Omega B.M.G-Plate reader. Trans-endothelial electrical resistance (TEER) was evaluated using the EVOM system, followed by quantifying gene expression of the endothelial tight junction (ETJs) using qPCR. **Results**: bEnd.3 cells had reduced cell viability after 72 h and 96 h exposure to MCF7CM under hypoxic and normoxic conditions. The ΔΨm in bEnd.3 cells were hyperpolarized after exposure to the hypoxic MCF7CM (*p* < 0.0001). However, the normoxic MCF7CM did not significantly affect the state of ΔΨm in bEnd.3 cells. ATP levels in bEnd.3 co-cultured with hypoxic and normoxic MCF7CM was significantly reduced (*p* < 0.05). The changes in brain endothelial mitochondrial activity were associated with a decrease in TEER of bEnd.3 monolayer co-cultured with MCF7CM under hypoxia (*p* = 0.001) and normoxia (*p* < 0.05). The bEnd.3 cells exposed to MCF7CM significantly increased the gene expression level of ETJs (*p* < 0.05). **Conclusions**: MCF7CM modulate mitochondrial activity in brain endothelial cells, affecting the brain endothelial barrier function.

## 1. Introduction

The Blood-brain barrier (BBB) is a multicellular barrier located between the blood and the brain tissues. It is composed of brain endothelial cells (BECs), followed by pericytes, basement membrane, and astrocytes [1]. BECs are characterized by the presence of continuous structural proteins called tight junctions (TJs). The TJs link the BECs together, thus, significantly limiting the paracellular flux of solutes and the passage of cells [2]. Moreover, TJs at the cerebral endothelium are only encountered in the brain capillaries and not at the systemic capillaries [1]. It has been hypothesized that the BBB may be disrupted by metastatic cancers such as breast cancer [3]. Breast cancer is the most prevalent malignant tumor in women and the second-leading cause of brain metastases following lung cancer [4]. Approximately 30% of women with metastatic breast cancer acquire brain metastases [5]. The initial step of cancer cells to metastasis is to separate from the primary tissue and enter the circulatory system. Then, the cancer cells are arrested at the capillary bed before the extravasation, which is followed by cancer cell proliferation at the new location [6]. For metastasis into the brain, breast cancer cells must cross the BBB [7]. Cancer cells commonly penetrate the endothelial barriers either by the transcellular or paracellular route. The transcellular route includes cancer cells moving in large vacuoles through endothelial cells [4,8], while the paracellular route requires a breakdown of the endothelial cell-cell junction to allow cancer cells to enter the brain [5]. Cancer cells manage to survive despite the exposure to the high stress of being in circulation [6,9]. At the point that the cancer cells are arrested at the BEC’s apical surface, and before penetrating the endothelial barrier [8], potential interactions occur between cancer cells and endothelial cells. These interactions are characterized by either physical adhesion to the BECs or via secretory paracrine factors [10,11], which induces disruption of the brain endothelial cells and facilitates the migration of cancer cells into the brain. The mechanism proposed for this interaction, in general, suggest that physiological changes occur in endothelial cells under the stimulation of cancer cells, resulting in the activation of adhesion molecules such as E-selectin in endothelial cells. Once these BEC plasma membrane proteins interact with their ligand on the metastatic cancer cell, a series of signaling pathways affect the opening of the cell-cell junctions allowing cancer cells to cross the endothelial barrier [12,13].

Interaction between endothelial cells and metastatic cancer cells induces genetic modification in the endothelial cells [14,15]. The physical interaction between metastatic cancer cells and endothelial cells occurs as an endothelial response to cancer stimulation [13]. The mediators of this stimulation are thought to be paracrine factors secreted from cancer cells [16]. Therefore it is not surprising that cancer cells release a variety of molecules such as growth factors, enzymes, and cytokines and that these molecules may modulate the endothelial properties [17,18]. Stimulated cancer cells secrete enzymes such as matrix-metalloproteinases, MMP2, and MMP9, which degrade the basement membrane components and tight junction proteins of BECs [19,20,21]. Cytokines can activate BECs by inducing the adhesion proteins such as E-selectin and P-selectin, followed by the presentation of vascular cell adhesion molecule-1 and intercellular adhesion molecule-1 at the surfaces of endothelial cells. These proteins are the mediators of the diapedesis of cancer cells [6]. Cancer cells induce changes in the endothelial cell by upregulating the expression of adhesion molecules receptors, remodelling the cytoskeleton fibres, and disrupting the endothelial cell-cell junctions [17]. The present study aimed to investigate the paracrine effect of breast cancer cells on the mitochondrial activity and viability of brain endothelial cells under both normoxic and hypoxic conditions.

## 2. Materials and Methods

### 2.1. Cell Culture & Condition Media Collection

Murine brain microvascular endothelial cells (bEnd.3 ATCC^®^ CRL-2299, Gaithersburg, MD, USA) and invasive breast cancer cells (MCF7(ATCC HTB-22)) were cultured in Dulbecco’s Modified Eagle Medium (DMEM. Gibco. No. 22320022, 8717 Grovemont Cir, Gaithersburg, MD, USA) supplemented with 10% Fetal Bovine Serum (FBS. Biowest. No. 10493-106, 2 Rue du Vieux Bourg, Nuaillé, France) and 100 U/mL penicillin/streptomycin (Gibco. No. 15070063) (Complete DMEM media). TrybleE (ThermoFisher Scientific, No. A1285901, 168 Third Avenue, Waltham, MA, USA) was used for harvesting the cells.

To collect the MCF7 conditioned medium (MCF7CM), 1 × 10^5^ of cancer cells were grown in 75-cm^2^ culture flasks in normal humidity 5% CO_2_ incubator at 37 °C, when they reached 50% confluence, the spent growth media was replaced with a fresh growth media and further incubated under normoxic conditions (21% O_2_) or hypoxic conditions (5% O_2_) for 48 h (The incubation under hypoxic conditions was performed by placing the TC flasks in a sterilized Modular Incubator Chamber (Hypoxia chamber—MIC 101)—Billups-Rothenberg, Inc., Sorrento Valley Blvd, San Diego, CA, USA). The hypoxia chamber is provided with a Greisinger Oxygen meter with a sensor (GOX 100-0-CO. No. 600437), which allowed for the measurement of O_2_ during the incubation). After 48 h incubation in either normoxic or hypoxic conditions, the supernatant was collected in ice-cooled centrifuge tubes, centrifuged at 3500 rpm for 5 min at 4 °C, then filtered with a GVS filter (0.20 µm). The liquated supernatants were collected in 2–5 mL tubes and stored at −80 °C until required.

### 2.2. bEnd.3 Treatments with MCF7CM

The collected supernatants were thawed at 21 °C and added to fresh complete DMEM to concentrations 20%, 40%, and 75%, called MCF7 conditioned media (MCF7CM). The bEnd.3 monoculture cells were seeded at the seeding densities required by the assay and incubated at normal humidity 5% CO_2_ incubator at 37 °C for 24 h to allow for cell attachment. Following 24 h incubation, the spent growth media were removed, then cells were exposed daily to either 20%, 40%, or 75% MCF7CM for 24, 48, 72, and 96 h.

Metastatic breast cancer cells, gain access to the CNS by crossing the endothelium of brain capillaries. These breast cancer cells firstly have to adhere to the apical/luminal side of the BEC, and then secondly, modulate/compromise the BECs via paracrine factors to cross the BBB. To therefore understand how paracrine factors from the luminal side of the capillary could affect the mitochondrial aspects of BEC function, bEnd.3 cells in Transwell inserts (Transwell^®^ insert (pore size of 0.45μm, filtration diameter of 12 mm, and an effective surface area of 0.6 cm^2^) (Merck-Millipore. PIHA01250, 6 Hatters Ln, Watford, UK) were treated by adding MCF7CM to the apical insert chamber on a daily basis. However, in bEnd.3 cell and MCF7 cell coculture experiments, the Transwell three-legged insert was used as the basis of our bicameral chamber experimental system, where MCF7 cells were seeded in the abluminal side (in the wells) while bEnd.3 cells were seeded in the inserts (apical chamber) to ensure that we could measure TEER across bEnd3 cell monolayers. These experiments were carried out in triplicate.

### 2.3. Research Design

**Figure 1:**
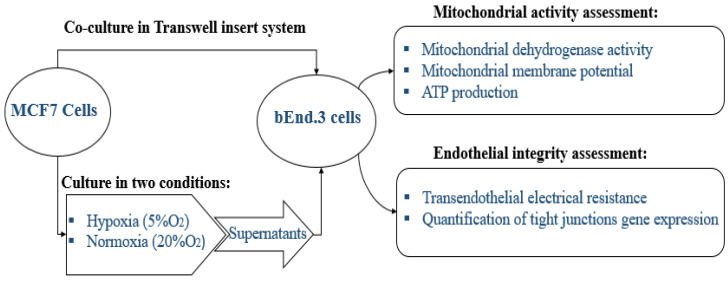
Schematic diagram of the research design to study the effect of the paracrine factors secreted by MCF7 cells on bEnd.3 cells.

The study is designed to study the physiological changes in brain endothelial cells bEnd.3 under the influence of MCF7 cells. All experiments were carried out in triplicate as a minimum (*n* = 3) and duplicated to ensure repeatability. The effect of normoxic and hypoxic paracrine factors secreted from MCF7 was compared. The diagram below in Figure 1 summarizes the research design of this study.

For the in vitro model of the BBB, we utilized the bicameral chamber system, where the well assumes the abluminal side of the capillary endothelium, while the apical chamber (the insert) assumes the luminal side of the capillary. This allowed for MCF7 cells to be cocultured in wells while a three-legged insert (Merck-Millipore, PIHA01250, 6 Hatters Ln, Watford, UK) was used for growing the endothelial BEC monolayers. These three-legged inserts also facilitated the movement of inserts between varying treatment conditions (e.g., normoxic versus hypoxic conditions).

In order to ensure that our MCF7CM always had sufficient metabolic constituents, a minimum of 25% fresh growth media (DMEM) was always added to the MCF7CM to make up the selected treatment concentrations (20%, 40%, and 75%). Furthermore, MCF7 cell cultures were grown to 50% confluency and exposed for maximally 48 h, before supernatants were removed and prepared for experimentation. Lastly, media for treating cells were replaced daily to ensure continuity in our treatment process.

In addition, we had to avoid using an O_2_ level that would compromise the viability bEnd.3 cells when cocultured under hypoxic conditions. We show in our study that bEnd.3 cell monolayers are indeed sensitive to 5% O_2_ but can recover within 24 h to control levels of permeability (TEER) (Figures 6 and 7).

### 2.4. Endothelial Cells Viability Assay

For the determination of cell viability, bEnd.3 cells were seeded on Transwell^®^ insert (pore size of 0.45μm, internal insert diameter of 12 mm, and an effective surface area of 0.6 cm^2^) (Merck-Millipore. PIHA01250, 6 Hatters Ln, Watford, UK) a density of 2000 cells/insert; the inserts were placed in 24 well plates (Bio-Smart Scientific. No. 30024, Park Edge Mews, Edgemead, Link Way, Edgemead, Cape Town, South Africa). Growth media (completed DMEM) was added to both luminal (300 µL) and basolateral side (800 µL), and cells were incubated at 37 °C and 5% CO_2_ overnight, allowing them to attach to the surface of the filter membrane. MCF7 cells were separately seeded at a density of 1000 cells/well on the same day in 12 well plates (Bio-Smart Scientific. No. 30012, Park Edge Mews, Edgemead, Link Way, Edgemead, Cape Town, South Africa).

Following 24 h of incubation, bEnd.3 cells were co-cultured with MCF7 cells by placing the inserts with bEnd.3 cells in the 12 well plates containing MCF7 cells. Plates were incubated at 37 °C with 5% CO_2_ for 96 h. Then, the inserts were removed from the 12 well plates and placed in new 24 well plates. This ensured that only bEnd.3 cells were assayed for viability. 100 µL of XTT solution was added to each insert, and the cells were incubated for an additional 4 h at 37 °C and 5% CO_2_. Then the medium from the inserts was transferred into 96 well plates (SPL-Life Sciences. No. 30096, 26, Geumgang-ro 2047 beon-gil, Naechon-myeon Pocheon-si, Gyeonggi-do, Korea). The absorbance was measured at 450 nm by using a microplate reader.

In addition, the bEnd.3 cells were seeded in 96-well plates (SPL-Life Sciences. No. 30096) at a density of 4 × 10^3^ cells/well, each experimental group was seeded in 5 replicate wells, the cells were incubated for 24 h and treated with MCF7CM as previously described (i.e., MCF7CM was added in the luminal compartment). Following the treatment, the viability of bEnd.3 cells were determined using an XTT assay kit (Roche. No. 11465015001) in 24 h intervals up to 96 h. At each 24 h interval, 50 µL of XTT solution was added to each well, the cells were then incubated for 4 h at 37 °C in a 5% CO_2_ incubator. The absorbance was then measured at 450 nm using a (Polarstar Omega B.M.G. Labtech, Allmendgrün 8, Ortenberg, Germany) microplate reader.

### 2.5. Mitochondrial Membrane Potential (∆ψm)

Changes in ∆ψm in bEnd.3 cells after exposure to MCF7CM were analyzed using Tetramethylrhodamine Ethylesterperchlorate (TMRE) (Thermofisher. No. T669) assay. TMRE is a permeable cationic, lipophilic dye, emitting red-orange fluorescent. It is actively taken up by active mitochondria into the negatively charged mitochondrial matrix. The intensity of the fluorescent signal obtained is indicative of the ∆ψm. The higher membrane potential (more polarized) indicates more TMRE accumulation in the mitochondrial matrix [17]. Therefore, the higher red-orange fluorescence would indicate a higher membrane potential (hyperpolarization). In this assay, the bEnd.3 cells were seeded in flasks at a density of 3 × 10^4^ per flask and treated as previously described. In addition, bEnd.3 were also treated with carbonylcyanide-3-chlorophenylhydrazone (CCCP) (Sigma, Eschenstr. 5, Taufkirchen, Germany) as a control. The CCCP is well known as an uncoupling agent of the ∆ψm. CCCP was used to confirm that the uptake of the TMRE is related to the mitochondrial membrane potential. At 24 h intervals, 100 µM of CCCP was added and incubated for 10 min before staining with Tetramethylrhodamine Ethylesterperchlorate (TMRE) (ThermoFisher Scientific, No. T669, 168 Third Avenue, Waltham, MA, USA) at 300 nM for 20 min. The solution stain was then removed, and cells were washed twice with PBS. Cells were then scraped and lysed in a lysis buffer composed of SDS (0.1% *v/v*) in 0.1M Tris-HCl buffer. The 150 µL of the lysates were loaded in 96 well plates. The fluorescence of TMRE was measured with a multi-well fluorescence plate reader with excitation and emission set at 508 ± 20 nm and 589 ± 40 nm, respectively. A total protein concentration was then determined in the remaining lysate samples corresponding in their lysates using Bicinchoninic acid (BCA) kit (ThermoFisher Scientific, No. 232225, address: 168 Third Avenue, Waltham, MA, USA). The fluorescence in each well was normalized for the protein concentration of its corresponding lysate.

### 2.6. ATP Production

The bEnd.3 were seeded on Transwell^®^ insert (pore size of 0.45 μm, filtration diameter of 12 mm, and an effective surface area of 0.6 cm^2^) at a density of 2 × 10^3^ cells/insert; the inserts were placed in 24 well plates. Growth media (completed DMEM) was added to both luminal (300 µL) and basolateral side (800 µL), cells were incubated at 37 °C and 5% CO_2_ overnight. MCF7 cells were separately seeded at a density of 1 × 10^3^ cells/well on the same day in 12 well plates. After 24 h, the growth medium was replaced with fresh completed DMEM (1 mL). Following 24 h incubation, bEnd.3 were placed in the 12 well plates which had MCF7 cancer cells growing in the well bottom. The co-culture cells were incubated at 37 °C under 5% CO_2_ for 96 h (During the co-culture incubation time, the medium in the inserts (for bEnd.3 cells) were changed daily). The inserts were then removed from 12 well plates and placed in new 24 well plates (this ensured that ATP detection solution will only apply on bEnd.3 cells). Then, 100µL of ATP detection solution was added to each insert. Cells were then incubated for an additional 5 min at room temperature in a plate shaker. The mixture from the inserts was transferred into 96 white well plates (SPL-Life Sciences. No. 31396, address: 26, Geumgang-ro 2047 beon-gil, Naechon-myeon Pocheon-si, Gyeonggi-do, Korea). The luminescence was measured using a microplate reader (B.M.G Labtech, address: Allmendgrün 8, Ortenberg, Germany).

In addition, bEnd.3 cells were seeded (1 × 10^3^ cells/well) in 96 white well plates (SPL-Life Sciences. No. 31396) and were exposed daily to the MCF7CM as previously described. At 24 h intervals, relative intracellular ATP levels were determined using the Mitochondrial ToxGlo™ kit (Promega (G8000), address: 2800 Woods Hollow Road, Madison, WI, USA). The Mitochondrial ToxGlo™ assay was conducted according to the supplier’s protocol. An ATP detection solution was prepared by mixing 10 mL of ATP buffer with an ATP detection substrate. The components were homogenized by vortex, forming an ATP detection solution. At 24 h intervals, 100 µL of ATP detection solution was added to each well. Then the plates were incubated for 5 min at room temperature in a plate shaker. ATP content was measured using a luminescent plate reader (B.M.G Labtech, Allmendgrün 8, Ortenberg, Germany).

### 2.7. Transendothelial Electrical Resistance (TEER)

The endothelial monolayer integrity was tested by determining the transendothelial electrical resistance (TEER) using the EVOM TEER measurement system. The bEnd.3 cells were grown on insert membrane (Transwell^®^ Inserts with 0.45 µm pore size) (Merck-Millipore. PIHA01250, 6 Hatters Ln, Watford, UK) at a density of 5 × 10^4^ cells/insert. MCF7 cells were seeded in the 12 well plates. Both cell lines were first grown separately for 96 h, then the inserts with bEnd.3 cells were placed in the 12 well plates where MCF7 cells were grown. The co-cultured cells were incubated in normoxic conditions, TEER was measured daily starting from day two.

In addition, bEnd.3 cells and MCF7 cells were grown separately in similar conditions for 72 h, then the two cell lines were co-cultured by placing the bEnd.3 inserts in the MCF7 well plates. The co-cultured cells were incubated in normoxic conditions (21% O_2_) for 24 h, then incubated in hypoxic conditions (5% O_2_) for the rest of the experimental time frame. TEER was measured daily starting from day three.

To determine the effect of MCF7CM on bEnd.3 monolayer transendothelial electrical resistance (TEER), bEnd.3 cells were seeded on inserts (Merck-Millipore. PIHA01250) at a density of 5 × 10^4^ cells/well in 12 well plates for 72 h and incubated with hypoxic and/or normoxic MCF7CM at 20%, 40%, and 75% concentrations. Cells were then incubated at 37 °C and 5% CO_2_. The TEER measurement started by day three. In control groups, bEnd.3 cells were grown in inserts and placed in wells with fresh media. The measurement was performed by connecting the electrodes to either side of the cell monolayer and measuring the resistance. The resistance of the blank (inserts without cells) was subtracted from the resistance value of the cell monolayer (inserts with cells), and the resultant value was multiplied by the surface area of the inserts to standardize the TEER value. The measurement was performed as described by Srinivasan et al., 2015 [22].

### 2.8. Quantitative PCR (q-PCR) Gene Expression Assay

To determine whether the reduction in the endothelial resistance of bEnd.3 cells exposed to MCF7CM was associated with the expression of tight junction proteins, a qPCR was performed to quantify the gene expression of tight junction proteins (Occludin and Claudin-5). The bEnd.3 cells were grown in T75 flasks and chronically exposed to normoxic or/and hypoxic MCF7CM as previously mentioned. Following the exposure, a total RNA was extracted using Tripure isolation reagent (Roche, Ref:11667157001, Ground Floor Liesbeeck House River Park, River Lane, Mowbray, Cape Town, South Africa). The first strand of cDNA was synthesized from total RNA using Transcriptor first-strand cDNA synthesis kit (Roche, No. 04379012001). The resultant cDNA served as a template for real-time PCR amplification using SYBR Luna universal qPCR master mix kit (New England bio labs), and Real-Time PCR System (Applied bio-system real-time-PCR instrument (ThermoFisher Scientific, REF 4484643). To amplify a fragment of claudin-5, occludin, and GAPDH (as house-keeping gene), the primer pairs detailed were used (Table 1). The amplification was conducted at 95 °C for 1 s, followed by 44 cycles of 95 °C for 15 s, 63 °C for 30 s and 95 °C for 15 s. Results were analyzed using the Pfaffl method as described by Pfaffl et al., 2002 [23].

### 2.9. Statistical Analysis

Statistical analysis was performed by Graph Pad Prism software (version 6), Data was expressed as mean ± SEM, and the differences between groups were analyzed by unpaired Students’ *t*-test or one-way ANOVA followed by Dunnett’s multiple comparison test. The significant level was accepted at *p* < 0.05 for a 95% confidence interval.

## 3. Results

### 3.1. The Effect of MCF7 Cells and Their Conditioned Media on bEnd.3 Cell Viability

**Figure 2:**
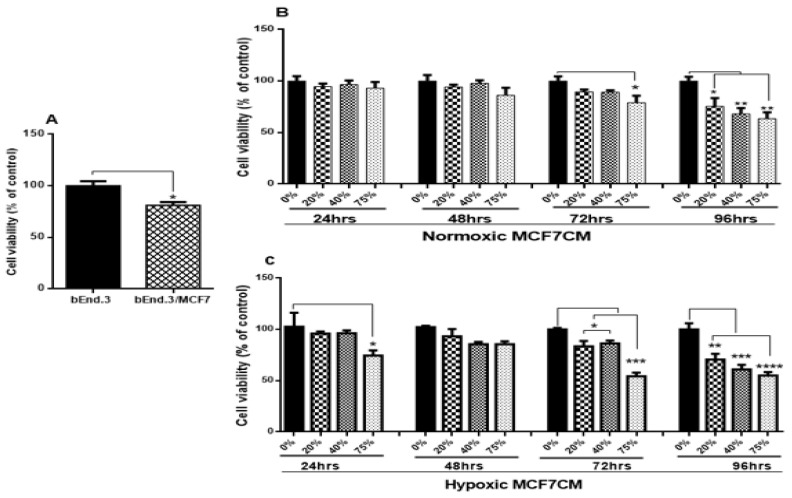
The viability of bEnd.3 cells under the chronic influence of MCF7 cells: (**A**) shows the effects of co-culture with MCF7 cells on bEnd.3 cell viability; (**B**) represents the viability of bEnd.3 cells after being treated daily with selected concentrations of MCF7CM produced from MCF7 cells under normoxic incubation (21% O_2_); and (**C**) shows the viability of bEnd.3 cells after exposure to selected concentrations of MCF7CM obtained from MCF7 cells under hypoxic conditions (5% O_2_). (*n* = 5), (* *p* < 0.05, ** *p* < 0.01, *** *p* < 0.001, **** *p* < 0.0001).

To evaluate how MCF7 cells modulate brain endothelial physiological functions, the viability of bEnd.3 cells co-cultured with MCF7 cells was evaluated using a XTT assay. As shown in Figure 2A, the viability of bEnd.3 cells co-cultured with MCF7 cells was significantly reduced (*p* < 0.01). Similar results were obtained after bEnd.3 cells were exposed to selected concentrations of MCF7 conditioned media (MCF7CM). Figure 2B shows that bEnd.3 cells, exposed to selected concentrations of MCF7CM under normoxic conditions for 24 h and 48 h, were not significantly different in their viability compared to the control. However, following 48 h of exposure to MCF7CM, the bEnd.3 cell viability was reduced after exposure to 75% for 72 h (*p* < 0.02), whereas at 96 h, MCF7CM exposure significantly reduced bEnd.3 cell viability in a dose-response manner (* *p* < 0.05, ** *p* < 0.01). The viability of bEnd.3 cells were significantly reduced after 24 h exposure to the media composed of 75% of hypoxic MCF7CM (* *p* = 0.0450). At 24 h, no significant difference was observed in the viability of bEnd.3 cells were exposed to 20% or 40% of hypoxic MCF7CM for the same point of time (Figure 2C). Although there was a decrease in the means of all concentrations of hypoxic MCF7CM, these were not statistically different to controls. However, the viability of bEnd.3 cells was significantly decreased at all concentrations of hypoxic MCF7CM following 72 h and 96 h exposure (* *p* < 0.05, ** *p* < 0.01, *** *p* = 0.0002, **** *p* < 0.0001).

### 3.2. The Effect of MCF7 CM on the Mitochondrial Membrane Potential (ΔΨm)

**Figure 3:**
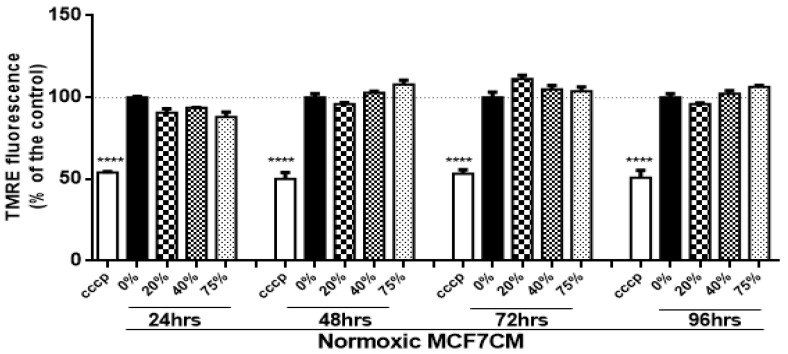
Shows the changes in mitochondrial membrane potential (ΔΨm) in bEnd.3 cells after daily exposure to MCF7CM cultured under normoxic conditions (20% O_2_). (CCCP: carbonylcyanide-3-chlorophenylhydrazone (negative control)). (*n* = 4) (****p < 0.0001).

The changes to mitochondrial membrane potential were investigated as a further measure of bEnd.3 endothelial function after MCF7CM exposure, using Tetramethylrhodamine Ethylesterperchlorate (TMRE) as a measure the ΔΨm. The functionality of the TMRE assay on bEnd.3 cells were carried out using carbonylcyanide-3-chlorophenylhydrazone (CCCP), a known inhibitor of ΔΨm (negative control). Treatment with CCCP decreased ΔΨm consistently throughout the study. Figure 3 illustrates that daily exposure of normoxic MCF7CM did not statistically affect the ΔΨm in bEnd.3 cells over 96 h.

**Figure 4:**
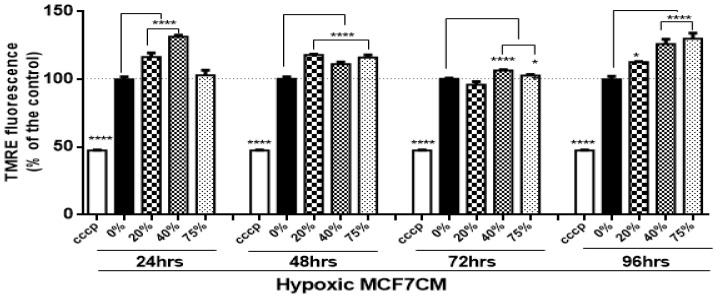
Shows the changes in mitochondrial membrane potential (ΔΨm) in bEnd.3 cells after chronic exposure to selected concentrations of MCF7CM derived from MCF7 cells cultured under hypoxic conditions (5% O_2_). (CCCP: carbonylcyanide-3-chlorophenylhydrazone (negative control)). (*n* = 4) (* *p* < 0.05, **** *p* < 0.0001).

Interestingly, bEnd.3 cells exposed to hypoxic MCF7CM show an increase in the ΔΨm at all concentrations compared with the control (Figure 4) (* *p* < 0.05, **** *p* < 0.0001). Exceptions to the statistical increase of ΔΨm occurred at 24 h at 75% and 72 h at 20% of bEnd.3 cells treated with hypoxic MCF7CM media.

### 3.3. ATP Level in bEnd.3 Cells under the Influence of MCF7 Cells

**Figure 5:**
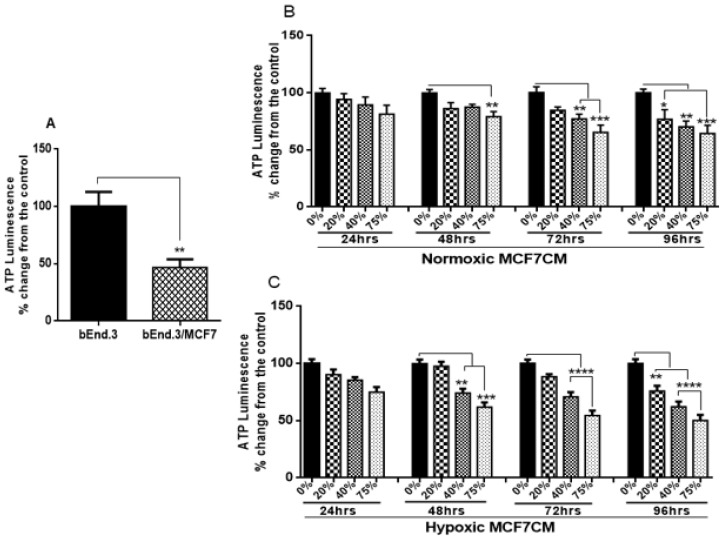
Shows the changes in cellular ATP level in bEnd.3 cells under the influence of MCF7 cells: (**A**) represents ATP levels in bEnd.3 cells co-cultured with MCF7cells; (**B**) represents ATP levels in bEnd.3 cells exposed to selected concentrations of MCF7CM produced from MCF7 cells cultured under normoxic conditions (21% O_2_); and (**C**) represents cellular ATP levels in bEnd.3 cells exposed to selected concentrations of MCF7CM derived from MCF7 cells cultured under hypoxic conditions (5% O_2_). (*n* = 4), (** *p* < 0.01, *** *p* < 0.001, **** *p* < 0.0001).

The classic function of mitochondria is to generate ATP through oxidative phosphorylation. Here, bEnd.3 cells co-cultured with MCF7 cells exhibited low levels of ATP production compared with the control (Figure 5A) (*p* < 0.001). Similar results were observed when bEnd.3 cells were subjected to both normoxic and hypoxic derived MCF7CM (Figure 5B,C). Exposure to normoxic MCF7CM (Figure 5B) caused a depression of ATP production. At 24 h and partly at 48 h, although the means of ATP produced were depressed, no statistical difference was obtained between the exposed cells and the control, except at 48 h exposure to 75% concentration, where ATP levels were significantly reduced (*p* < 0.0001). After 72 h exposure, only bEnd.3 cells exposed to 40% and 75% demonstrated a decrease in ATP level relative to the control (*p* < 0.01, and *p* < 0.001 respectively). At 96 h exposure, the ATP productivity of bEnd.3 cells were significantly reduced at all concentrations compared to controls (see Figure 5B: * *p* < 0.05, ** *p* = 0.003, *** *p* < 0.001).

Although bEnd.3 cells exposed to hypoxic MCF7CM showed similar effects to bEnd.3 cells exposed to normoxic MCF7CM, they were more pronounced. At 24 h exposure all means were depressed and there were no statistical differences between hypoxic MCF7CM treatment and controls. At 48 h and 72 h, only cells exposed to 40% and 75% concentration show a significant decrease in ATP level. However, at 96 h exposure, ATP concentrations were decreased for all treatments (Figure 5C).

### 3.4. The Effect of MCF7 Co-Culture on Transendothelial Electrical Resistance (TEER) of Confluent bEnd.3 Cell Monolayers under Normoxic Conditions

**Figure 6:**
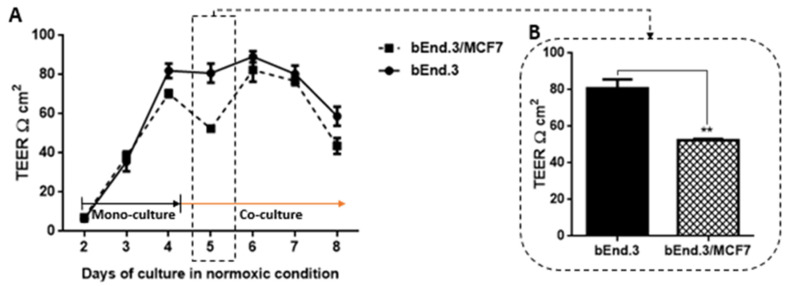
Transendothelial electrical resistance (TEER) of bEnd.3 monolayers cocultured with MCF7 cells under normoxia (21% O_2_): (**A**) shows the changes in TEER across confluent monocultured bEnd.3 cells after co-culture with MCF7 cells under normoxic conditions; (**B**) TEER of bEnd.3 monolayers after 24 h co-culture with MCF7 cells. Day five was the only day that showed a significant difference from controls. (*n* = 3), (** *p* = 0.002).

The permeability across monolayers of bEnd.3 cells cultured as a monoculture (control) or co-culture (with MCF7cells) used the Transwell bicameral system to measure TEER. As shown in Figure 6A the TEER across bEnd.3 cell confluent monolayers were measured in control groups and experimental groups daily for eight days. For days two to four, both groups were grown as a mono-culture, and no difference was observed in TEER. By the end of day four, mono-cultures of confluent bEnd.3 monolayers were introduced to co-cultured of MCF7 cells. TEER of mono-cultured confluent bEnd.3 monolayers compared to co-cultured confluent bEnd.3 monolayers were significantly reduced (*p* < 0.01) after the co-cultures of MCF7 cancer cells (see Figure 6B). Interestingly, the reduction of TEER was not constant, as bEnd.3 cells co-culture with MCF7cells recovered their TEER by day six, although the increase in TEER remained non-statistically lower than the control.

### 3.5. The Effect of MCF7 Co-Culture on Transendothelial Electrical Resistance (TEER) of Confluent bEnd.3 Cell Monolayers under Hypoxic Conditions

**Figure 7:**
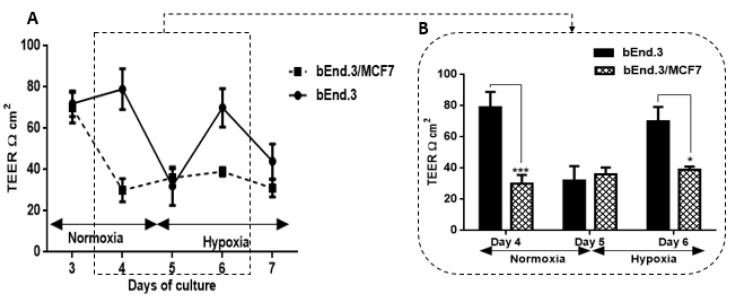
Transendothelial electrical resistance (TEER) of bEnd.3 monolayers cocultured with MCF7 cells under hypoxia (5% O_2_): (**A**) shows changes in TEER across bEnd.3 monolayers co-cultured with MCF7 cells in normoxia and hypoxia. Note that mono-cultures of bEnd.3 monolayers are transiently affected by hypoxic conditions, but recover to control levels within 24 h; (**B**) show changes in the TEER across bEnd.3 cells monolayers co-cultured with MCF7 cells under normoxic and hypoxic incubation. (* *p* < 0.05, *** *p* < 0.001), (*n* = 3).

In addition, TEER was measured for bEnd.3 cell confluent monolayers cultured as a monoculture under normoxic conditions and then introduced co-cultured MCF7 cells under hypoxic conditions (Figure 7). Similar to the normoxic condition, bEnd.3 cells in both groups were cultured as a monoculture until day three.

TEER measurement was monitored from day three and before the co-culture of bEnd.3 monolayers with MCF7cells. Until day four, cells were incubated under normoxic conditions (Figure 7A). By the end of day four, both bEnd.3 monolayers monocultured and co-cultured with MCF7 were incubated under hypoxic conditions until day seven. As shown in Figure 7A, under normoxia, the co-culture with MCF7 cells significantly decreased the endothelial resistance (see Figure 7B on day four (*** *p* < 0.001)). The reduction in TEER of co-cultured bEnd.3 monolayers were not changed by placing the cells in hypoxia. Interestingly, the resistance of monocultured bEnd.3 monolayers (control) also decreased under hypoxic conditions (see Figure 7A on day five). However, the TEER of the control recovered to normal levels by day six (see Figure 7A,B on day six) before declining again on day seven. In contrast, TEER of bEnd.3 co-cultured with MCF7 under hypoxic conditions remained suppressed from day four until the end of the experiment and was always statistically lower than the monocultured bEnd.3 monolayers.

### 3.6. The Effect of MCF7CM on the TEER across Confluent bEnd.3 Monolayers

**Figure 8:**
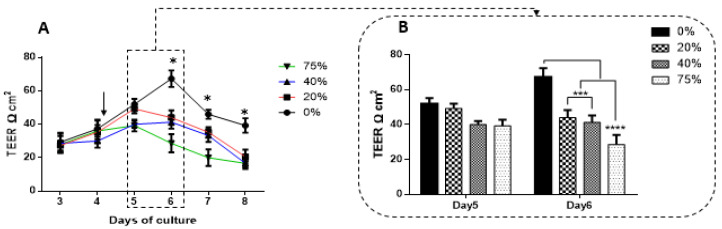

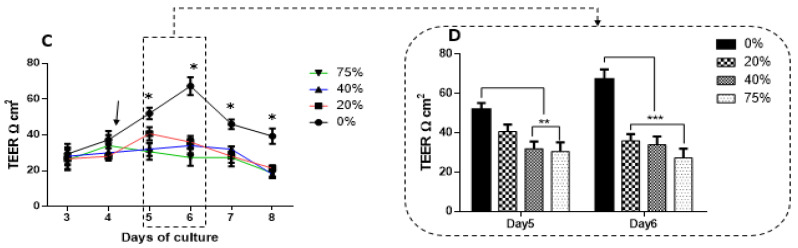
TEER of bEnd.3 cells exposed to selected concentrations of MCF7CM: (**A**) represents TEER across bEnd.3 monolayers exposed to selected concentrations of MCF7CM media harvested from MCF7 cells cultured under normoxic conditions (21% O_2_); the arrow indicates the start of the exposure to normoxic MCF7CM media. The asterisks (*) refers to the significant difference between controls (0%) and cells exposed to selected concentration of normoxic MCF7CM (** *p* < 0.01, *** *p* < 0.001, *** *p* < 0.001); (**B**) shows the peak effect of normoxic MCF7CM media on TEER across treated bEnd.3 monolayers after 24 h (day five) and 48 h (day six) treatment (*** *p* < 0.001, **** *p* < 0.0001); (**C**) shows TEER of bEnd.3 monolayer exposed to selected concentrations of hypoxically derived MCF7CM media. The arrow in Figure 8C indicates the start of the exposure to hypoxically derived MCF7CM media. The asterisks (*) refers to a significant difference between controls (0%) and cells exposed to normoxic MCF7CM (* *p* < 0.05); and (**D**) shows the peak effects of TEER between control (0%) and the exposed cells after 24 h (day five) and 48 h (day six) exposure to hypoxically derived MCF7CM media (** *p* < 0.01, *** *p* < 0.001), (*n* = 3).

In addition, the exposure to MCF7CM increased the permeability of the bEnd.3 monolayers (Figure 8A,B). Confluent bEnd.3 monolayers exposed to normoxic MCF7CM demonstrated lower resistance compared to controls (Figure 8A). The exposure to normoxic MCF7CM started by the end of day four of cell culture, and by day five TEER decreased non-statistically compared to the control TEER (0%) (Figure 8B in day five). Following 48 h of MCF7CM exposure, both “peak” TEER (in Figure 8B block) and subsequent TEER were significantly decreased at all concentrations (see Figure 8B on day six). However, the exposure to hypoxic derived MCF7CM significantly reduced TEER of bEnd.3 monolayers after 24 h exposure (Figure 8C), particularly at 40% and 75% (see Figure 8D: day five). On day six, both “peak” TEER and subsequent TEER was significantly suppressed compared to the control (Figure 8D day six).

### 3.7. qPCR-Gene Expression Analysis

**Figure 9:**
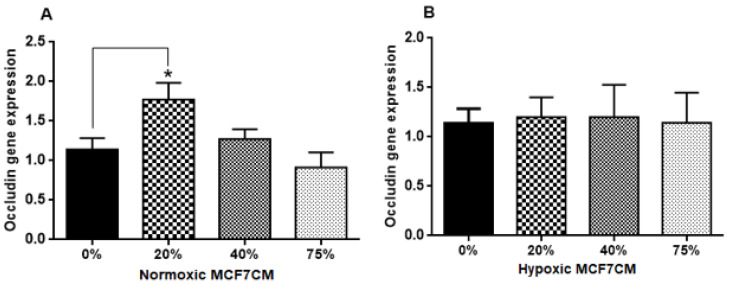
Gene expression level of Occludin in bEnd.3 cells after the exposure to selected concentrations of MCF7CM produced under normoxia (**A**) and hypoxia (**B**). (* *p* = 0.0123), (*n* = 4).

The level of mRNA expression for Occludin and Claudin-5 was analyzed from bEnd.3 cells exposed to selected concentrations of MCF7CM media. cDNA was transcripted from mRNA and processed using qPCR. Results demonstrated that Occludin gene expression in confluent layers of bEnd.3 cells exposed to normoxic MCF7CM media increased compared to the control (Figure 9A), particularly after exposure to 20% of normoxic MCF7CM. However, gene expression levels of Occludin were not changed in the bEnd.3 cells after exposure to hypoxic MCF7CM (Figure 9B).

**Figure 10:**
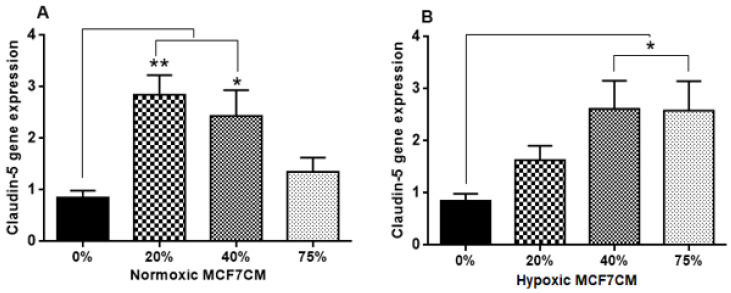
Gene expression level for Claudin-5 in bEnd.3 cells after the exposure to selected concentrations of MCF7CM media derived under normoxic (**A**) and hypoxic (**B**) conditions. (* *p* < 0.05.** *p* = 0.002), (*n* = 4).

Claudin-5 gene expression increased after exposure to both normoxic and hypoxic derived MCF7CM media (Figure 10A, B). The treatment with 75% normoxic MCF7CM media did not produce a statistical difference in Claudin-5 gene expression compared with the control (Figure 10A), whereas, 20% and 40% of normoxic MCF7CM significantly elevated Claudin-5 gene expression (** *p* < 0.01, * *p* < 0.05 respectively). It was interesting to note that the treatment profile of both Occludin and Claudin-5 to normoxic MCF7CM, were similar. bEnd.3 cells exposed to the higher concentrations of hypoxic derived MCF7CM (40% & 75%) showed significantly higher levels of Claudin-5 gene expression than the control (Figure 10B)) (* *p* < 0.05).

## 4. Discussion

Metastatic breast cancer often relocates across the notoriously protective BBB to bring about its pathological effects in the neural tissue of the central nervous system (CNS) [3]. We used the established in vitro brain endothelial model of BBB, using the BEC-line (bEnd.3), to further investigate some of the underlying mechanisms used by breast cancer cells (MCF7) to induce the physiological compromise of the endothelial cells of brain capillaries. Furthermore, the phenomenal rate at which metastatic cancer tumors develop most times leads to zones of hypoxia within the tumor itself, as there is often a lag time to angiogenesis within the tumor. We wanted to further understand whether tumor cells act differently under conditions of normoxia (21% O_2_) and under conditions of low O_2_ (5% O_2_), or hypoxic conditions, and if the level of paracrine cross-talk between cancer cells BECs of brain capillaries are affected.

To facilitate these experiments, we used the established bicameral experimental setup to measure the paracrine effects of MCF7 cells grown as a co-culture with bEnd.3 cells. Secondly, to study whether these paracrine secretions had a dose-related effect on the BECs, we collected the supernatant of MCF7 cells grown under normoxic and hypoxic conditions (MCF7CM media) and treated BECs by exposing them on a daily basis.

### 4.1. bEnd.3 Cell Viability after MCF7 Exposure

Cell viability is a measure of the proportion of live, healthy cells within a cultured-cell population, and it is essentially used to evaluate the response of a population to a treatment or an agent [24]. Cell viability is dependent on various cellular functions such as enzyme activity, cell membrane permeability, mitochondrial activity, and ATP generation [24,25]. In the present study, cell viability was determined by measuring the activity of mitochondrial dehydrogenases in response to treatment with various concentrations of supernatant derived from breast cancer cells (MCF7 cells) using the XTT cell viability assay. Mitochondrial dehydrogenases catalyze the reactions of transferring the protons in the electron transfer chain, which in turn drives ATP synthesis [26]. We wanted to evaluate if the viability of BECs exposed to paracrine factors from these MCF7 cells would be affected. Our results showed a marked reduction in cell viability after prolonged exposure of bEnd.3 cells to cocultured MCF7 cells (Figure 2A), and MCF7CM generated under normoxic (Figure 2B), and hypoxic (Figure 2C) conditions. The effects of bEnd.3 exposed to hypoxic MCF7CM was much more pronounced than bEnd.3 cells exposed to normoxic MCF7CM. Furthermore, our data indicate that cell viability was not affected within the 48 h, and whereas only the highest concentration (75%) of normoxic MCF7CM depressed viability at 72 h, all concentrations suppressed BEC viability by treatment with hypoxic MCF7CM. The delayed response in viability to MCF7CM treatment also indicated that the process whereby endothelial cells are affected is manifested after several iterations of cell division. Furthermore, although there is a similar profile between normoxic and hypoxic derived MCF-7CM, the more pronounced effects under hypoxic conditions suggests that breast cancer cells accelerate and increase their paracrine secretory effects on endothelial viability.

A study by Strilic et al. (2016) showed that cancer cells induced endothelial necroptosis in vitro and in vivo, which appears to be essential for the process of extravasation and metastasis of cancer cells [27]. Previous studies have shown that cancer cells under hypoxic conditions demonstrated both genetic and metabolic alteration [28,29,30], which promote their malignancy [31,32]. In view of the fact that cancer paracrine factor secretion has been reported to be different in normoxia and hypoxia [33], this might explain the difference in endothelial response against the stimulation with hypoxic and normoxic MCF7CM. Constant viability reduction of bEnd.3 cells exposed to MCF7 cells and the derived MCF7CM is an indicator that MCF7 paracrine exposure has the ability to impact mitochondrial function in a dose-related manner and that these effects are more pronounced when exposed to hypoxic-derived MCF7CM, indicates that under hypoxic conditions MCF7 cells scale-up their secretion of paracrine factors to induce long-term suppression of capillary brain endothelial cell viability.

### 4.2. Mitochondrial Activity of bEnd.3 Cells after MCF7 Exposure

#### 4.2.1. Mitochondrial Membrane Potential (ΔΨm)

The suppressive effects of MCF7CM on the levels of mitochondrial dehydrogenase (viability) of bEnd.3 cells, motivated us to investigate the changes in the mitochondrial membrane potential (ΔΨm). Physiologically, during oxidative phosphorylation, the proton pumps (Complexes I, III, and IV) transport hydrogen ions (positive charges) across the mitochondrial inner membrane, resulting in the mitochondrial membrane potential (ΔΨm). This process is essential for generating a proton gradient which in turn drives the production of ATP [34]. In this process, an accumulation of H^+^ in the outer membrane space subsequently flows down an H ^+^ -proton gradient back into the mitochondria via the ATP-producing F1/F0 ATP-synthase to complete the electron transport chain and drive the generation of ATP.

In the present study, bEnd.3 cells exposed to a selected concentration of MCF7CM show an increase in the mitochondrial membrane potential in comparison to the control. At the mitochondrial level, an accumulation of TMRE (positive charge) in the mitochondrial matrix yields high fluorescent intensity proportional to the ΔΨm polarization potential. The hyperpolarized state is a result of the transfer and accumulation of H^+^ in the outer mitochondrial intermembrane space. Mitochondrial hyperpolarization was more evident in bEnd.3 treated with hypoxic MCF7CM, presenting mitochondrial hyperpolarization after 24 h exposure to hypoxic MCF7CM (Figure 4), whereas bEnd.3 cells exposed to normoxic MCF7CM did not show a significant difference in TMRE fluorescent relative to the control (Figure 3). Mitochondrial hyperpolarization can occur as a result of the inhibition of ATPase activity. ATPase inhibition reduces the electrochemical gradient utilization, which results in less ATP generation and ΔΨm hyperpolarization [35,36]. Given the scope of the study, we only investigated the state of ΔΨm as an indicator for mitochondrial activity. However, further study is suggested to determine the mechanism of mitochondrial hyperpolarization in brain endothelial cells in response to exposure of cancer cells.

#### 4.2.2. ATP Generation

Given the hyperpolarization of the BEC mitochondria exposed to hypoxically derived MCF7CM, we investigated the levels of ATP in treated BECs. The main function of mitochondria is ATP synthesis. Therefore, ATP generation is an accurate indicator of mitochondrial activity and cell viability [37]. Mitochondria are the major source of ATP generation (through oxidative phosphorylation) [38], with mitochondria yielding 36 ATP, compared to glycolysis (yielding only 2 ATP per glucose molecule) [39]. Cerebral endothelial cells possess more mitochondria than systemic endothelial cells, indicating the vital role of ATP in the regulatory processes across the BBB [40]. Dysfunction in endothelial mitochondria induces BBB disruption in vitro and in vivo [41]. In the present study, the investigation of ATP generation in brain endothelial cells under the influence of cancer was necessary. Therefore, bEnd.3 cells, chronically exposed to both MCF7 cells and their MCF7CM, were monitored for their ATP production using a luminescence assay. Results show a marked reduction of ATP generation in bEnd.3 cells co-cultured with MCF7 cells (Figure 5A) or exposed to normoxic (Figure 5B) or hypoxic MCF7CM (Figure 5C). The decrease in ATP level was observed after 48 h of exposure to 75% of MCF7CM. Taken together with the hyperpolarization of the ΔΨm after exposed to hypoxic MCF7CM, suppressed ATP levels confirm that MCF7 cells induce changes in the mitochondria activity in brain endothelial cells which might contribute in the BBB dysfunction. The hyperpolarization of the outer intermembrane space of the mitochondria suggests that one of the MCF7 derived paracrine affectively decouples H^+^ gradient into the matrix of the mitochondria and thereby suppresses the production of ATP.

### 4.3. Transendothelial Electrical Resistance (TEER) of bEnd.3 Monolayers after Exposure to MCF7 Co-Culture

Endothelial cells are the functional site of the BBB [42]. They are connected by tight junctions, forming a barrier against the paracellular diffusion of molecules and ions into the brain [43]. Occludin and Claudin-5 are the most prominent proteins which form the brain endothelial tight junctions [44,45]. The expression of these proteins is critical for brain capillary (endothelial) integrity, and downregulation of these proteins decreases the brain endothelial resistance [46]. The present study demonstrated that TEER across monolayers of brain endothelial cells (bEnd.3) transiently decreased over a period of 36 h (Figure 7) after exposure to cocultured breast cancer cells (MCF7). This finding is supported by observations that showed that brain endothelial cells were induced in the presence of MCF7 cells to enhance the transmigration of breast cancer through the endothelium instead of providing a secure barrier against breast cancer cells [47]. Interestingly, although the decrease in the endothelial resistance under the treatment of normoxic MCF7CM was not constant, endothelial cells recovered after 24 h to control levels of transendothelial resistance (Figure 6A). This window of transient decreased TEER may indeed assist the breast cancer cells by providing a window for migration across the BBB. We also investigated whether co-culturing MCF7 cells with bEnd3 cells under hypoxic conditions (5% O_2_) would exacerbate the increased transient permeability (decreased TEER) observed during co-culture under normoxic conditions. Hypoxic conditions transiently decreased our control bEnd3 monolayers for 24 h, but these monolayers adapted within 24 h to normal control levels (Figure 7A). Under normoxic conditions, TEER decreased transiently and recovered to control levels (Figure 6A). However, in hypoxia cocultured bEnd.3 cells, this recovery was nullified by hypoxic conditions (Figure 7). The recovery of TEER of the control bEnd.3 cell monolayer within 24 h was in contrast to the bEnd.3 cell monolayer co-cultured with MCF7 cells, where continued hypoxia-induced long-term suppression of TEER over next 48 h (Figure 7: days six and seven). It is, therefore, indicative that hypoxia causes paracrine induced continued suppression of TEER (increased transendothelial permeability). It is well known that cancer cells modulate their metabolism to adapt to hypoxic conditions, and thus the mechanism to disrupt the endothelial barrier may differ under hypoxic and normoxic conditions. Moreover, metastatic breast cancer secretes enzymes including matrix metalloproteinases [48], which are involved in the degradation of tight junction proteins [20]. The transient low TEER after exposure to hypoxia (Figure 7B) by bEnd.3 cells not co-cultured with MCF7 cells (control), which recovered to normal levels of TEER the next day, endorses recent reports that hypoxia can induce increase permeability (low TEER) in brain endothelial cells [49]. These conditions for hypoxia may be induced in the capillary by the arrest of circulating cancer cells to the apical membranes of brain capillary endothelia, thereby decreasing lumen effective diameter and reducing blood flow (which induces local hypoxic conditions for endothelial cells).

### 4.4. Transendothelial Electrical Resistance of bEnd.3 Monolayers after Exposure to MCF7CM

The recording of TEER across most monolayers typically follows a biphasic response where TEER initially increases to a peak, plateaus and then gradually decreases. We compared peak TEER responses on days five and six after exposing bEnd3 monolayers with selected concentrations of MCF7CM under normoxic (21% O_2_) and hypoxic conditions (5% O_2_) (Figure 8). Under both normoxic and hypoxic MCF7CM treatment, peak TEER was suppressed. However, the suppression under normoxic derived MCF7CM was not statistically different from controls on day five, whereas already on day five hypoxic derived MCF7CM suppressed TEER significantly. It is clear that both normoxic and hypoxically derived MCF7CM induces a dose-response effect on TEER and that hypoxic conditions for cancer cells aggressively upscales this effect, producing effects on target capillary endothelia at a much faster rate and also more pronounced effect. However, the similar profiles of these effects suggest that mechanism whereby MCF7 cells deploy to affect changes on TEER occur via a similar molecular mechanism, and thus demands further investigation.

Although there is a clear dichotomy between treating bEnd.3 monolayers apically (luminal perspective) and coculturing MCF7 cells with bEnd3 cells from an abluminal perspective, a number of significant aspects must be noted: Firstly, our data indicate that these paracrine factors can produce similar effects on BECs, whether they are applied from the luminal or abluminal side of endothelial monolayers; and secondly, it speaks to the permeability of these paracrine factors with regards to the ease at which they cross the plasma membrane of the cells.

### 4.5. The Effect of MCF7CM Exposure on the Tight Junctions (TJs) Expression

In this study, the TEER data (Figure 6, Figure 7 and Figure 8) did not correlate with the gene expression of the tight junctions, Occludin and Claudin-5 (Figure 9 and Figure 10, respectively). Normoxically derived MCF7CM had similar effects on both Occludin and Claudin-5 expression with increasing concentration of MCF7CM resulting in dose-related suppression in gene expression relative to treated samples. Relative to controls, only the lowest concentration of normoxically derived MCF7CM produced an elevated expression of Occludin. Surprising, hypoxically derived MCF7CM had no statistical effect relative to controls. However, bEnd.3 cells which were exposed to hypoxically derived MCF7CM, significantly produced an elevated expression of Claudin-5. Increased levels of Claudin-5 is usually correlated with increased TJs proteins at the apico-lateral membrane of endothelial cells, with the concomitant increase in TEER. However, our data always produced a decrease in TEER after exposure to MCF7CM. Thus, MCF7CM based paracrine factors may be involved in the prevention of the incorporation of TJ proteins at the apico-lateral membranes or the mechanism by which cancer secretions decrease the endothelial resistance is not mediated by the tight junction at the paracellular route, but via increased transcellular permeability resulting in the decrease in TEER. In view of reports that activated cancer cells release various types of metalloproteinase enzymes, which depredate the tight junction proteins [21], suggest that cancer cells would prefer to compromise paracellular spaces and in so doing provide a passage for metastasic cancer cells into the brain parenchyma. PCR reflects the early protein expression components in the process of mRNA translation to a primary protein. However, before this primary protein is inserted into the membrane, the protein undergoes numerous post-translational modification steps before it is finally is inserted into the membrane along with being attached to ZO molecules which connects the TJ protein to actin scaffolding proteins in the cytoplasm of the cell. We suspect that the paracrine factors secreted by the MCF-7 cells compromise one or several of these post-translational aspects compromising the insertion and/or the functional aspects of the TJ-proteins. We have seen in our lab that bEnd.3 cell confluent monolayers produce immunofluorescence to both Occludin and Claudin-5 (unpublished data), so we were surprised that our results did not show clear effects on mRNA transcription/translational processes for Occludin and Claudin-5. A limitation to the PCR data is that we do not have collaborating western blot data and immunofluorescent data, which may produce clues as to where MCF-7 paracrine factors are producing their effects on TJ proteins. Nevertheless, this requires additional investigation.

Furthermore, the less pronounced effects of MCF7CM on Occludin compared to Claudin-5 mRNA expression suggest that breast cancer cells target the Claudin-5 TJs pathways rather than Occludin. This postulate is supported by the fact that the tightness of paracellular spaces is dependent on Claudin-5 rather than Occludin [45].

## 5. Conclusions

Our study showed that breast cancer cells can affect brain capillary endothelial cells by modulating the function of mitochondria, leading to the decrease of generation of ATP with the concomitant effects of causing decrease cell viability, increase paracellular permeability by modulating especially Claudin-5 TJ expression. These induced effects on the brain endothelial capillary cell function show how breast cancer cells use paracrine factors to induce access into brain tissue. Our data show that hypoxic conditions produce exacerbated and accelerated effects on brain capillary endothelial cells. The dose-related responses of MCF7CM indicate that if the secretion of paracrine factors can be minimized, it could also decrease the ability of breast cancer cells to cross the BBB and metastasize CNS tissue. A low level of O_2_ is considered as normoxia in some literature [31], and our results show that just by reducing O_2_ concentration, breast cancer cells can trigger a more aggressive state. Since the research scope of this study was in vitro, and due to the limitation of the in vitro experiments (particularly in co-culture studies), the repeatability of the study in an in vivo system is recommended. One of the important outcomes of this study is the responsiveness of this brain endothelial cell line (bEnd3) to MCF7 co-culture and the treatment of MCF-7CM conditioned media. This supports our construct of an in vitro BBB model on which we can extensively study the effects of cancer cells on various physiological variables. These findings may help to develop new therapeutic targets to prevent cancer metastasis to the brain.

## Figures and Tables

**Table 1:** Primer sequences for quantitative PCR (qPCR) amplification of complementary DNA (cDNA). GAPDH: Glyceraldehyde phosphate dehydrogenase.

N	Primers	Primer Pairs (Sequence (5′ > 3′))	Product Length	°C
1	GAPDH	Forward: AGGAGAGTGTTTCCTCGTCCC	199	63
		Reverse: TGCCGTTGAATTTGCCGTGA		
2	Claudin-5	Forward: CCCAGTTAAGGCACGGGTAG	126	53–63
		Reverse: GGCACCGTCGGATCATAGAA		
3	Occludin	Forward: TTTCAGGTGAATGGGTCACCG	242	63
		Reverse: ACTTTCAAAAGGCCTCACGGA		

## Data Availability

All experimental data collected is archived within the University of the Western Cape (UWC) archives and is available as per UWC data and intellectual property policy guidelines and its associated copyright protection.

## References

[1] Serlin Y., Shelef I., Knyazer B., Friedman A. (2015). Anatomy and physiology of the blood–brain barrier. Semin. Cell Dev. Biol..

[2] Kadry H., Noorani B., Cucullo L. (2020). A blood–brain barrier overview on structure, function, impairment, and biomarkers of integrity. Fluids Barriers CNS.

[3] Witzel I., Oliveira-Ferrer L., Pantel K., Müller V., Wikman H. (2016). Breast cancer brain metastases: Biology and new clinical perspectives. Breast Cancer Res..

[4] Woodward J. (2008). Crossing the endothelium. Cell Adhes. Migr..

[5] Herman H., Fazakas C., Haskó J., Molnár K., Mészáros Á., Nyúl-Tóth Á., Szabó G., Erdélyi F., Ardelean A., Hermenean A. (2019). Paracellular and transcellular migration of metastatic cells through the cerebral endothelium. J. Cell. Mol. Med..

[6] Labelle M., Hynes R.O. (2012). The initial hours of metastasis: The importance of cooperative host–tumor cell interactions during hematogenous dissemination. Cancer Discov..

[7] Reymond N., D’Agua A.B.B., Ridley A. (2013). Crossing the endothelial barrier during metastasis. Nat. Rev. Cancer.

[8] Haskó J., Fazakas C., Molnár K., Mészáros Á., Patai R., Szabó G., Erdélyi F., Nyúl-Tóth Á., Győri F., Kozma M. (2019). Response of the neurovascular unit to brain metastatic breast cancer cells. Acta Neuropathol. Commun..

[9] Strilic B., Offermanns S. (2017). Intravascular survival and extravasation of tumor cells. Cancer Cell.

[10] Wirtz P., Konstantopoulos D., Searson K. (2012). Mechanical forces in metastasis. Biomol. Eng..

[11] Leong H., Robertson A.E., Stoletov K., Leith S.J., Chin C.A., Chien A.E., Hague M.N., Ablack A., Carmine-Simmen K., McPherson V.A. (2014). Invadopodia are required for cancer cell extravasation and are a therapeutic target for metastasis. Cell Rep..

[12] Lambert A.W., Pattabiraman D., Weinberg R.A. (2017). Emerging biological principles of metastasis. Cell.

[13] Li W., Khan M., Mao S., Feng S., Lin J.-M. (2018). Advances in tumor-endothelial cells co-culture and interaction on microfluidics. J. Pharm. Anal..

[14] Khodarev N.N., Yu J., Labay E., Darga T., Brown C.K., Mauceri H.J., Yassari R., Gupta N., Weichselbaum R.R. (2003). Tumour-endothelium interactions in co-culture: Coordinated changes of gene expression profiles and phenotypic properties of endothelial cells. J. Cell Sci..

[15] Hoffmann O.I., Ilmberger C., Magosch S., Joka M., Jauch K.-W., Mayer B. (2015). Impact of the spheroid model complexity on drug response. J. Biotechnol..

[16] Wilhelm I., Molnár J., Fazakas C., Haskó J., Krizbai I.A. (2013). Role of the blood-brain barrier in the formation of brain metastases. Int. J. Mol. Sci..

[17] Mierke C.T. (2008). Role of the endothelium during tumor cell metastasis: Is the endothelium a barrier or a promoter for cell invasion and metastasis?. J. Biophys..

[18] Shenoy A.K., Lu J. (2014). Cancer cells remodel themselves and vasculature to overcome the endothelial barrier. Cancer Lett..

[19] Feng S., Cen J., Huang Y., Shen H., Yao L., Wang Y., Chen Z. (2011). Matrix metalloproteinase-2 and -9 secreted by leukemic cells increase the permeability of blood-brain barrier by disrupting tight junction proteins. PLoS ONE.

[20] Rempe R.G., Hartz A.M.S., Bauer B. (2016). Matrix metalloproteinases in the brain and blood–brain barrier: Versatile breakers and makers. J. Cereb. Blood Flow Metab..

[21] Roomi M.W., Kalinovsky T., Rath M., Niedzwiecki A. (2017). Modulation of MMP-2 and MMP-9 secretion by cytokines, inducers and inhibitors in human glioblastoma T-98G cells. Oncol. Rep..

[22] Srinivasan B., Kolli A.R., Esch M.B., Abaci H.E., Shuler M.L., Hickman J.J. (2015). TEER measurement techniques for in vitro barrier model systems. J. Lab. Autom..

[23] Pfaffl M.W. (2002). Relative expression software tool (REST(C)) for group-wise comparison and statistical analysis of relative expression results in real-time PCR. Nucleic Acids Res..

[24] Kamiloglu S., Sari G., Ozdal T., Capanoglu E. (2020). Guidelines for cell viability assays. Food Front..

[25] Stockert J.C., Blázquez-Castro A., Cañete M., Horobin R.W., Villanueva Á. (2012). MTT assay for cell viability: Intracellular localization of the formazan product is in lipid droplets. Acta Histochem..

[26] Romani A.M. (2018). Physiology and Pathology of Mitochondrial Dehydrogenases.

[27] Strilic B., Yang L., Albarran-Juarez J., Wachsmuth L., Han K., Müller K.H.U.C., Pasparakis L.W.M., Offermanns S. (2016). Tumour-cell-induced endothelial cell necroptosis via death receptor 6 promotes metastasis. Nature.

[28] Strese S., Fryknäs M., Larsson R., Gullbo J. (2013). Effects of hypoxia on human cancer cell line chemosensitivity. BMC Cancer..

[29] Al Tameemi W., Dale T.P., Al-Jumaily R.M.K., Forsyth N.R. (2019). Hypoxia-modified cancer cell metabolism. Front. Cell Dev. Biol..

[30] Nejad A.E., Najafgholian S., Rostami A., Sistani A., Shojaeifar S., Esparvarinha M., Nedaeinia R., Javanmard S.H., Taherian M., Ahmadlou M. (2021). The role of hypoxia in the tumor microenvironment and development of cancer stem cell: A novel approach to developing treatment. Cancer Cell Int..

[31] Muz B., de la Puente P., Azab F., Azab A.K. (2015). The role of hypoxia in cancer progression, angiogenesis, metastasis, and resistance to therapy. Hypoxia.

[32] Petrova V., Annicchiarico-Petruzzelli M., Melino G., Amelio I. (2018). The hypoxic tumour microenvironment. Oncogenesis.

[33] Eilertsen M., Pettersen I., Andersen S., Martinez I., Donnem T., Busund L.-T., Bremnes R.M. (2012). In NSCLC, VEGF—A response to hypoxia may differ between squamous cell and adenocarcinoma histology. Anticancer Res..

[34] Zorova L.D., Popkov V.A., Plotnikov E.Y., Silachev D.N., Irina B., Jankauskas S.S., Babenko V.A., Zorov S.D., Balakireva V., Juhaszova M. (2018). Mitochondrial membrane potential. Anal. Biochem..

[35] Gergely P., Grossman C., Niland B., Puskas F., Neupane H., Allam F., Banki K., Phillips P.E., Perl A. (2002). Mitochondrial hyperpolarization and ATP depletion in patients with systemic lupus erythematosus. Arthritis Rheum..

[36] Forkink M., Manjeri G.R., Liemburg-Apers D., Nibbeling E., Blanchard M., Wojtala A., Smeitink J.A., Wieckowski M., Willems P.H., Koopman W.J. (2014). Mitochondrial hyperpolarization during chronic complex I inhibition is sustained by low activity of complex II, III, IV and V. Biochim. Biophys. Acta (BBA) Bioenerg..

[37] Ian P., Lanza R., Sreekumaran K., Nair M.D. (2010). Mitochondrial metabolic function assessed in vivo and in vitro. Curr. Opin. Clin. Nutr. Metab. Care.

[38] Kühlbrandt W. (2015). Structure and function of mitochondrial membrane protein complexes. BMC Biol..

[39] Strickland M., Stoll E.A. (2017). Metabolic reprogramming in glioma. Front. Cell Dev. Biol..

[40] Wong B.W., Marsch E., Treps L., Baes M., Carmeliet P. (2017). Endothelial cell metabolism in health and disease: Impact of hypoxia. EMBO J..

[41] Doll D.N., Hu H., Sun J., Lewis S.E., Simpkins J.W., Ren X. (2015). Mitochondrial crisis in cerebrovascular endothelial cells opens the blood–brain barrier. Stroke.

[42] Sweeney M.D., Zhao Z., Montagne A., Nelson A.R., Zlokovic B.V. (2019). Blood-brain barrier: From physiology to disease and back. Physiol. Rev..

[43] Krouwer V.J.D., Hekking L.H.P., Langelaar-Makkinje M., Regan-Klapisz E., Post J.A. (2012). Endothelial cell senescence is associated with disrupted cell-cell junctions and increased monolayer permeability. Vasc. Cell.

[44] Liebner S., Kniesel U., Kalbacher H., Wolburg H. (2000). Correlation of tight junction morphology with the expression of tight junction proteins in blood-brain barrier endothelial cells. Eur. J. Cell Biol..

[45] Fisher D., Mentor S. (2020). Are claudin-5 tight-junction proteins in the blood-brain barrier porous?. Neural Regen. Res..

[46] Liu W.-Y., Wang Z.-B., Zhang L.-C., Wei X., Li L. (2012). Tight junction in blood-brain barrier: An overview of structure, regulation, and regulator substances. CNS Neurosci. Ther..

[47] Lee K.Y., Kim Y.-J., Yoo H., Lee S.H., Park J.B., Kim H.J. (2011). Human brain endothelial cell-derived COX-2 facilitates extravasation of breast cancer cells across the blood-brain barrier. Anticancer Res..

[48] Mehner C., Hockla A., Miller E., Ran S., Radisky D.C., Radisky E.S. (2014). Tumor cell-produced Matrix Metalloproteinase 9 (MMP-9) drives malignant progression and metastasis of basal-like triple negative breast cancer. Oncotarget.

[49] Luo P.-L., Wang Y.-J., Yang Y.-Y., Yang J.-J. (2018). Hypoxia-induced hyperpermeability of rat glomerular endothelial cells involves HIF-2α mediated changes in the expression of occludin and ZO-1. Braz. J. Med. Biol. Res..

